# The Protective Effect of Icariin on Mitochondrial Transport and Distribution in Primary Hippocampal Neurons from 3× Tg-AD Mice

**DOI:** 10.3390/ijms17020163

**Published:** 2016-01-27

**Authors:** Yijing Chen, Shuangxue Han, Xiuxian Huang, Jiazuan Ni, Xiaoyang He

**Affiliations:** 1 College of Life Science, Shenzhen Key Laboratory of Marine Bioresources and Ecology, Shenzhen University, Shenzhen 518060, China; lilyq335@126.com (Y.C.); shuangxuehan2012@163.com (S.H.); jzni@szu.edu.cn (J.N.); 2 College of Life Science, Shenzhen Key Laboratory of Microbial Genetic Engineering, Shenzhen University, Shenzhen 518060, China; huangxx@siat.ac.cn

**Keywords:** Alzheimer’s disease, icariin, mitochondrial transport, mitochondrial dysfunction

## Abstract

Icariin, a pharmacologically active component isolated from the Chinese herb Epimedium, has been shown to improve spatial learning and memory abilities in Alzheimer’s disease (AD) rats through inhibition of Aβ production and tau protein hyperphosphorylation. However, the potential mechanism of icariin-induced protective effects against mitochondrial dysfunctions in AD still remains unclear. In the present study, we investigated the effect of icariin on the modulation of mitochondrial transport and distribution in primary hippocampal cultures from triple-transgenic (3× Tg) AD mice. The results showed that icariin enhanced mitochondrial motility and increased mitochondrial index and mitochondrial length and size in the diseased neurons. Additionally, the expression of the key mitochondrial enzyme, pyruvate dehydrogenase-E1α (PDHE1α), and the post synaptic density protein 95 (PSD95), was preserved in AD neurons after icariin treatment, accompanied by a downregulation of Aβ and phosphorylated tau expression in the corresponding areas. Further study showed that icariin treatment resulted in a decrease in mitochondrial fission protein dynamin-related protein 1 (Drp1) and an increase in fusion protein Mitofusin 2 (Mfn2). These data indicate that icariin can promote mitochondrial transport, protect mitochondria against fragmentation and preserve the expression of mitochondrial and synaptic functional proteins in AD neurons. Thus, icariin may be a potential therapeutic complement for AD and other mitochondrial malfunction-related neuronal degenerative diseases.

## 1. Introduction

Alzheimer’s disease (AD) is one of the most commom neurodegenerative diseases and is characterized by cognitive decline, amyloid plaques, and neurofibrillary tangles [1]. Amyloid plaques are mainly composed of the amyloid-β peptide (Aβ), which is generated from the sequential cleavage of amyloid precursor protein (APP) by β-site APP cleavage enzyme 1 (BACE1) and γ-secretase [2]. It has been reported that the deposition of Aβ in mitochondria and synapses may cause dysfunctions in mitochondria and impair neuronal functions [3,4,5,6,7,8,9,10,11,12]. Deficits in the expression and activity of key mitochondrial enzymes such as pyruvate dehydrogenase (PDH) have been identified in post-mortem brain tissue from patients with AD and mouse models of AD [13,14,15,16]. PDH is the key rate-limiting enzyme in mitochondria to convert pyruvate into acetyl coenzyme A (acetyl-CoA) and initiate the tricarboxylic acid (TCA) cycle for energy generation [17]. The malfunction of this enzyme causes pyruvate accumulation and stimulates anaerobic metabolism to lactic acid.

Neurofibrillary tangles are composed of hyperphosphorylated forms of tau, which is a microtubule-associated protein [18]. In AD, tau is hyperphosphorylated and accumulates in neurons, where it forms paired, helical filaments. Hyperphosphorylated tau has been found to impair mitochondrial trafficking in axonal processes, leading to the abnormal mitochondrial dynamics and the aberrant distribution of mitochondria in AD-affected neurons [19,20,21,22]. Mitochondria are vital for supporting neuronal/synaptic activities. After being synthesized in the neuronal soma, synaptic mitochondria are then transported to dendrites and axons where they actively provide energy to fuel synaptic functions including neurotransmitter release and the maintenance of neuronal communication. Defects in synaptic mitochondria may ultimately compromise synaptic function and cause synaptic neurodegeneration [23,24,25,26]. Thus, searching for agents or drugs that are capable of sustaining normal distribution and trafficking of mitochondria may be important therapeutic strategies to prevent or delay neurodegeneration in AD.

Icariin (C_33_H_40_O_15_; molecular weight: 676.65, ICA) is a pharmacologically active component that was isolated from the Chinese herb Epimedium, which has been traditionally used as an aphrodisiac and antirheumatic remedy in Asia [27]. It has been reported that icariin administration improved spatial learning and memory abilities in rat models of AD, and the mechanisms may be related to its inhibitory effects on Aβ production and tau protein hyperphosphorylation [28,29,30,31]. However, the potential mechanism of icariin-induced protective effect against mitochondrial dysfunctions in AD still remains unclear. We recently reported that icariin treatment significantly recovered the learning-memory deficits in triple-transgenic (3× Tg) AD mice, and we found that icariin could enhance the level of brain metabolite *N*-acetylaspartate (NAA), increase the production of adenosine triphosphate (ATP) and the expression of mitochondrial proteins pyruvate dehydrogenase-E1α (PDHE1α) and cytochrome c oxidase IV (COX IV) in AD mouse brain, which reveals an important role of icariin in the regulation of mitochondrial metabolism against AD [32]. In the present study, we sought to determine the effects of icariin on the modulation of mitochondrial transport and distribution in primary hippocampal neurons from 3× Tg-AD mice. This transgenic (Tg) mouse model is advantageous for studying AD since it develops both plaques and tangles in an age-related manner, and thus it more closely mimics the neuropathology of the disease in humans compared to other mouse models [18].

## 2. Results

### 2.1. Characterization of Amyloid-β Peptide (Aβ) and Tau Expression in Primary Hippocampal Neurons from 3× Transgenic (Tg)-Alzheimer’s Disease (AD) Mice

**Figure 1:**
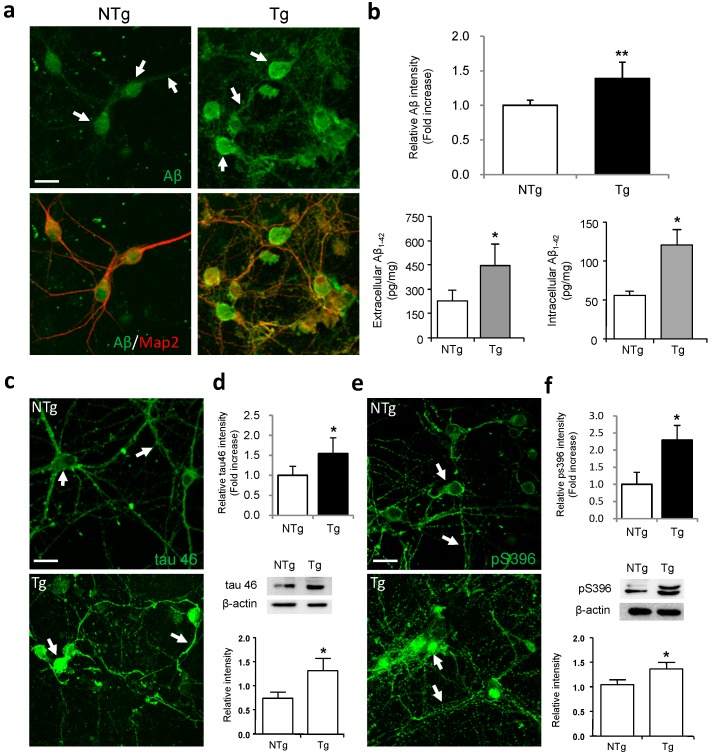
The overexpression of amyloid-β peptide (Aβ), tau and phosphorylated tau in 3× transgenic (Tg)-Alzheimer’s disease (AD) neurons. (**a**) Double-labeling analysis of neuronal marker microtubule-associated protein 2 (MAP2) with Aβ in neurons from nontransgenic (NTg) and Tg neurons. Tg cells showed an overexpression of Aβ in the soma and neurites as compared to NTg controls (white arrows); (**b**) Quantification revealed a significant difference in Aβ staining between Tg and NTg neurons. The enzyme linked immunosorbent assay (ELISA) demonstrated that the levels of extracellular and intracellular Aβ were higher in Tg neurons; (**c**) Total tau immunoreactivity in NTg and Tg neurons as measured with the tau 46 antibody. The immunoreactivity of tau 46 was more intense in the cell bodies and neuritic fibres of Tg neurons as compared to NTg controls (white arrows); (**d**) Quantification revealed a significant difference in tau 46 staining between Tg and NTg neurons. Western blot assay demonstrated that the level of total tau was significantly higher in Tg neurons; (**e**) Phosphorylated tau immunoreactivity in NTg and Tg neurons as measured with the phospho-tau (Ptau) antibody pS396. Tg neurons showed intense pS396 staining in the cytoplasm and neurites as compared to NTg controls (white arrows); (**f**) Quantification revealed a significant difference in pS396 staining between Tg and NTg neurons. Western blot assay demonstrated that the level of Ptau-396 was higher in Tg neurons. Bars graph (means ± SD) represented three independent experiments. * *p* < 0.05, ** *p* < 0.01. Scale bars in (**a**,**c**,**e**) = 20 μm.

In order to evaluate the effect of icariin on mitochondrial distribution and transport in AD-affected neurons, we first characterized the expression pattern of Aβ in hippocampal neurons from 3× Tg-AD mice, using an anti-Aβ_1–42_ antibody and a neuronal marker microtubule-associated protein 2 (MAP2) antibody. The nontransgenic (NTg) neurons that were cultured for 11 days *in vitro* (DIV) showed faint and evenly distributed Aβ expression in the somas and neuronal processes, whereas in Tg neurons, the Aβ immunoreactivity was more intense with some punctuated staining in the cell bodies and neurites (Figure 1a, white arrows). Quantitative analysis of Aβ staining revealed significant differences in the expression level of Aβ between Tg and NTg neurons (Figure 1b). To further examine the levels of extracellular and intracellular Aβ in AD neuronal culture, we performed the enzyme linked immunosorbent assay (ELISA). Our results showed that the levels of extracellular and intracellular Aβ were significantly higher in Tg neurons relative to NTg controls (*p* < 0.05) (Figure 1b). These data confirm that Aβ is overexpressed in this *in vitro* model [33].

Next, we characterized the expression patterns of tau and its phosphorylated isoforms in primary cultures of 3× Tg-AD neurons. We first examined the total tau expression by using the tau 46 antibody, which recognizes all six isoforms of the protein. At 11 DIV, NTg neurons showed moderate expression of tau 46 in the cell bodies and neuritic fibres (Figure 1c, white arrows). However, the immunoreactivity was more intense in Tg neurons, which had strong staining that covered the cell bodies, indicating overexpression of the protein. Next, we evaluated the expression pattern of tau phosphorylated at serine 396 using the antibody anti-tau pS396. As shown in Figure 1e, NTg neurons displayed weak staining in the cytoplasm and neurites, whereas Tg neurons showed intense pS396 staining that was distributed over the somas (white arrows). In agreement with the immunocytochemistry experiments, Western blot analysis showed higher levels of tau 46 and pS396 in Tg cells in comparison to that in NTg controls (*p* < 0.05) (Figure 1d,f).

### 2.2. Alterations in Synaptic and Mitochondrial Protein Expression in AD Neurons

**Figure 2:**
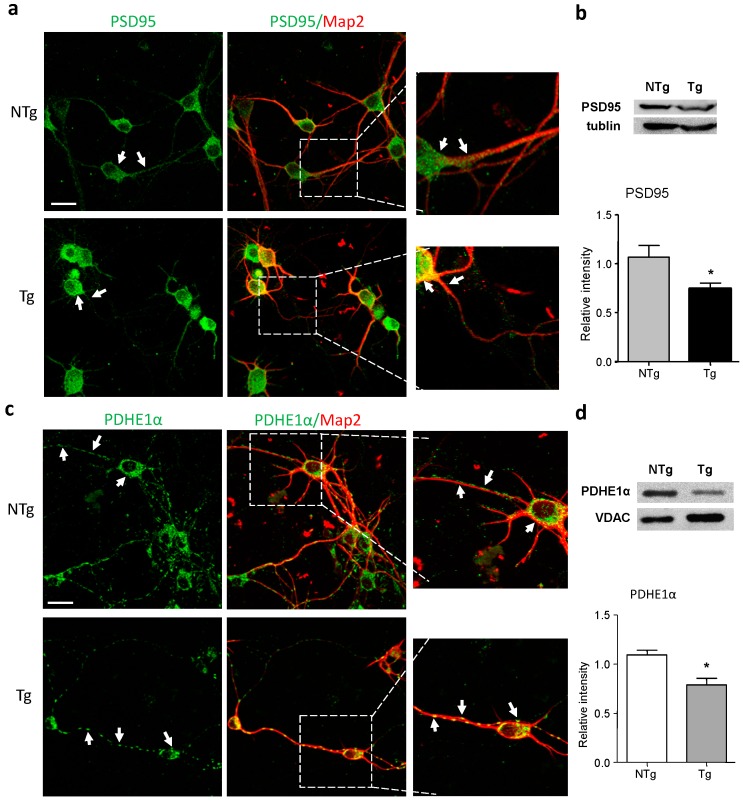
Altered expressions of synaptic and mitochondrial proteins in 3× Tg-AD neurons. Areas boxed in **a** and **c** are shown at higher magnification in the right panels, respectively. (**a**) Double-labeling analysis of neuronal marker MAP2 with post synaptic density protein 95 (PSD95) in neurons from NTg and Tg neurons. PSD95 expression was weaker in the neurites of Tg cells as compared to NTg group and restricted to the soma (white arrows); (**b**) Western blot assay demonstrated that the level of PSD95 was lower in Tg neurons; (**c**) Double-labeling analysis of MAP2 with pyruvate dehydrogenase-E1α (PDHE1α) in neurons from NTg and Tg neurons. PDHE1α expression was more evenly distributed in NTg cells whereas Tg cells showed fragmented expression of PDHE1α along the neuronal processes (white arrows); (**d**) Western blot assay demonstrated that the level of PDHE1α was lower in Tg neurons. Voltage-dependent anion-selective channel protein (VDAC) was used as a mitochondrial loading control. Bars graph (means ± SD) represented three independent experiments. * *p* < 0.05. Scale bars in (**a**,**c**) = 20 μm.

Given that Aβ and tau overexpression in AD neurons might affect synaptic development and cause synaptic degeneration, we then examined the expression of the post synaptic density protein 95 (PSD95) in primary neurons using immunostaining analysis. As a dendritic postsynaptic component of the neuron, PSD95 plays an important role in synaptic plasticity and signal transduction in the neuronal network and could be used as a postsynaptic marker of glutamatergic synapses [34]. As shown in Figure 2a, PSD95 displayed an abundant expression in the somas and neurites of NTg neurons, whereas in Tg neurons, PSD95 immunostaining was restricted to the cell bodies and fainter in the neuronal processes. Further examination using Western blot assay showed that the level of PSD95 was significantly lower in Tg neurons, in comparison to that in NTg controls (*p* < 0.05) (Figure 2b). These results indicate that synaptic deficits are present in primary neurons from 3× Tg-AD mice.

Mitochondria are essential for supplying energy to synapses to support neuronal functions, and thus synaptic dysfunctions are usually related to mitochondrial deficits. Therefore, we examined the expression of mitochondrial protein PDH (PDHE1α subunit), which is a key mitochondrial enzyme that is involved in the tricarboxylic acid cycle and energy production. PDHE1α is a mitochondrial inner membrane protein that is widely used as a mitochondrial marker. As shown by immunofluorescence analysis of PDHE1α and MAP2 antibodies, NTg neurons exhibited strong cytoplasmatic and neuritic expression of PDHE1α (Figure 2c). However, the staining was weaker and fragmented in neurites from the neurons of 3× Tg-AD mice relative to in NTg cells. Additionally, Western blot assay showed lower levels of PDHE1α in Tg cells in comparison to that in NTg controls (*p* < 0.05) (Figure 2d).

### 2.3. Icariin Preserved Mitochondrial Mass and Promoted Mitochondrial Transport

**Figure 3:**
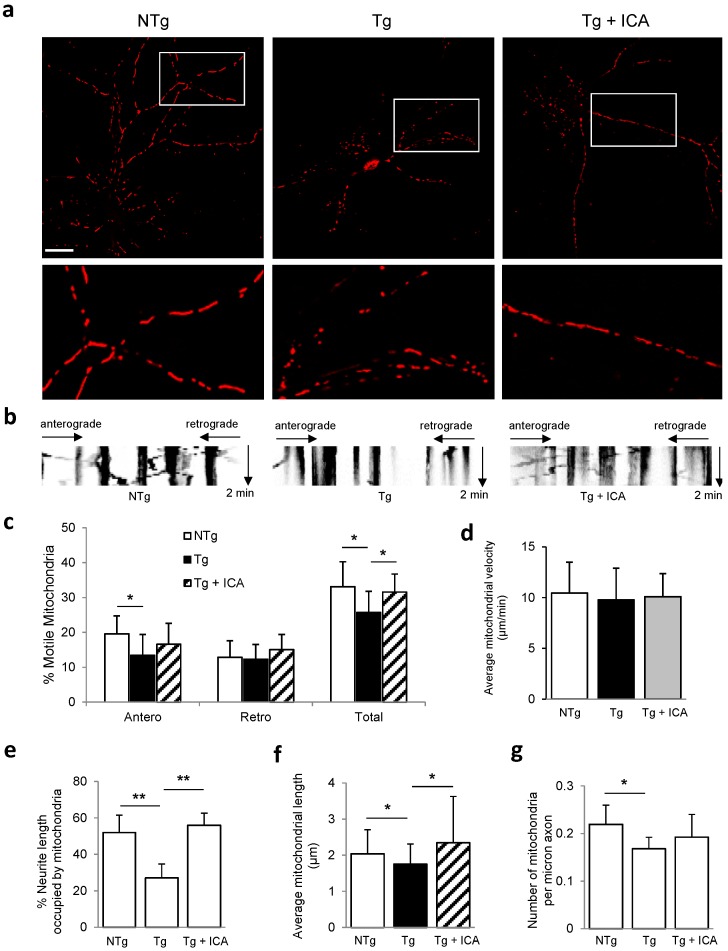
Effect of icariin on mitochondrial trafficking and distribution within neurites of 3× Tg-AD neurons. (**a**) DsRed-mito transfected hippocampal neurons from NTg, Tg and Tg + ICA groups were imaged. Representative images are shown in upper panels. Areas boxed in **a** are shown at higher magnification in the lower panels, respectively; (**b**) Representative kymograph images of the three experimental groups; (**c**) Percentages of anterograde-transported, retrograde-transported, and total movable mitochondria were calculated. Calculations were based on analysis of kymographs; (**d**) The average transport speed of movable mitochondria was calculated; (**e**) Axonal mitochondrial index (the proportion of neuritic length occupied by mitochondria) in neurons from NTg, Tg and Tg + ICA groups; (**f**) Mitochondrial length was increased in Tg neurons after icariin treatment; (**g**) Axonal mitochondrial density was evaluated as number of mitochondria per micron axon. Bar graphs (means ± SD) represented three independent experiments. * *p* < 0.05, ** *p* < 0.01. Scale bar in (**a**) = 20 μm.

The reduced and fragmented expression of PDHE1α in Tg neurons is suggestive of mitochondrial deficits in AD-affected neurons. Consistent with this result, we found that DsRed-labeled mitochondria appeared to be smaller and fragmented in Tg cells relative to in NTg controls. We then sought to investigate the effect of icariin on sustaining mitochondrial integrity. Previous study has demonstrated the protective effect of icariin on Aβ-induced cytotoxicity in primary neurons, which is dose dependent and shows the highest cell viability at the dosage of 20 μM icariin for 24 h [30]. In the present study, we found that treatment with icariin (20 μM) for 24 h could partially reverse the fragmentation of mitochondria in Tg neurons, revealing a protective effect of icariin against mitochondrial fragmentation in AD diseased neurons (Figure 3a).

To fulfill the synaptic roles in the neruonal cells with constant energy supply, mitochondria needs to travel from the soma to axon terminals. Thus, we monitored the trafficking of DsRed-labeled mitochondria within axonal processes. In comparion to the mitochondrial motility in the NTg control group, Tg neurons showed a significant decrease in the percentage of total motile mitochondria (NTg, 33.1% ± 7.1% *vs.* Tg, 25.7% ± 6.1%; *p* < 0.05) and in anterogradely moving mitochondria (NTg, 19.6% ± 5.1% *vs.* Tg, 13.4% ± 6.0%; *p* < 0.05) (Figure 3b,c and Supplementary Movies 1 and 2). However, we did not observe a significant alteration in the percentage of motile mitochondria moving retrogradely in Tg neurons relative to in NTg controls (NTg, 12.9% ± 4.7% *vs.* Tg, 12.3% ± 4.3%; *p* = 0.754). With icariin treatment, the percentage of total motile mitochondria in Tg cells increased to 31.6% ± 5.2% (*p* < 0.05 as compared with Tg control group), although the increases in anterogradely moving mitochondria (16.6% ± 6.0%, *p* = 0.227 as compared with Tg control group) and retrogradely moving mitochondria (15.0% ± 4.3%, *p* = 0.145 as compared with Tg control group) did not reach statistical significance (Supplementary Movie 3). Next, we measured the mitochondrial velocity in neuronal cultures. We did not observe a significant difference in the average speed of mitochondria between Tg and NTg neurons (NTg, 10.4 ± 3.1 µm/min *vs.* Tg, 9.8 ± 3.1 µm/min; *p* = 0.569), and icariin treatment did not significantly change the velocity of Tg cells compared to Tg control group (Tg + ICA, 10.1 ± 2.3 µm/min *vs.* Tg, 9.8 ± 3.1 µm/min; *p* = 0.459) (Figure 3d). These results indicate a dramatic reduction in the total movable mitochondria, especially in anterogradely-transported mitochondria in hippocampal neruons from Tg mice, and that icariin is able to promote the movement of mitochondria within neurites of AD diseased neurons.

To further evaluate the effect of icariin treatment on neuritic mitochondrial content, we measured the axonal mitochondrial index in primary neurons, which was calculated as the proportion of the neuritic length occupied by mitochondria. As shown in Figure 3e, Tg neurons exhibited a significant decrease in the mitochondrial index in comparison to that in NTg controls (NTg, 52.0% ± 9.5% *vs.* Tg, 27.1% ± 7.6%; *p* < 0.001), which was remarkably increased after treatment with icariin (Tg + ICA, 56.0% ± 6.6% *vs.* Tg, 27.1% ± 7.6%; *p* < 0.001).

Next, we investigated the changes in mitochondrial morphology by measuring mitochondrial length and density in axonal processes. There was a significant decrease in the average mitochondrial length in Tg neurons compared to in NTg neurons (NTg, 2.04 ± 0.67 µm *vs.* Tg, 1.75 ± 0.56 µm; *p* < 0.05) (Figure 3f). When Tg neurons were treated with icariin, the length of the mitochondria was significantly increased (Tg + ICA, 2.35 ± 1.28 µm *vs.* Tg, 1.75 ± 0.56 µm; *p* < 0.05). Axonal mitochondrial density was significantly decreased in Tg neurons relative to in NTg neurons (NTg, 0.22 ± 0.04 mitochondria/µm *vs.* Tg, 0.17 ± 0.02 mitochondria/µm; *p* < 0.05) (Figure 3g). Treatment with icariin slightly increased the mitochondrial density in Tg cells, but the difference was not significant (Tg + ICA, 0.19 ± 0.05 mitochondria/µm *vs.* Tg, 0.17 ± 0.02 mitochondria/µm; *p* = 0.114). Overall, we found that the mitochondrial length and size were greatly reduced in Tg neurons relative to the NTg neurons, and that icariin was able to increase the mitochondrial mass in Tg neurons mainly by enhancing the length rather than the number of mitochondria in the axonal processes.

### 2.4. Effects of Icariin on Mitochondrial Dynamics and Synaptic Protein Expression

**Figure 4:**
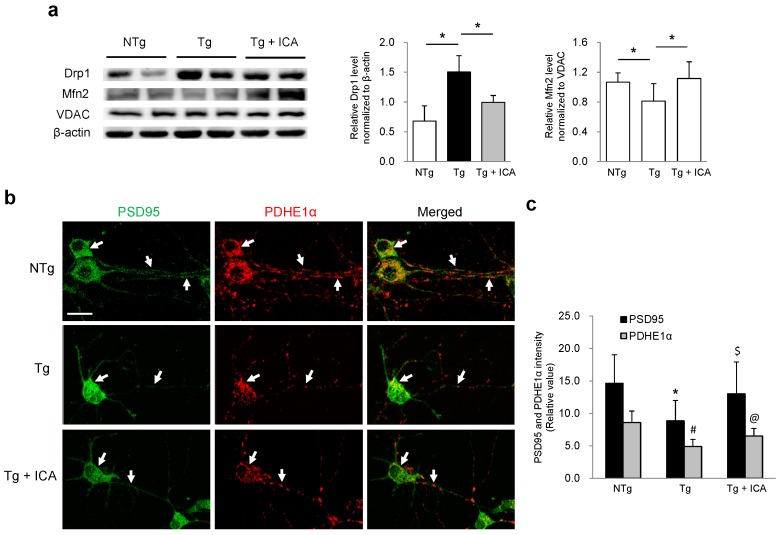
Effects of icariin on the modulation of mitochondrial dynamics and synaptic protein expression in 3× Tg-AD primary neuronal cells. (**a**) Western blot assay showed that icariin reduced the level of mitochondrial fission protein Drp1 and increased the level of mitochondrial fusion protein Mfn2 in Tg neurons; (**b**) Double-labeling analysis of PSD95 with PDHE1α in neurons from NTg, Tg and Tg + ICA groups. NTg neurons showed intense staining of PSD95 and PDHE1α in the cytoplasm and neurites. PSD95 was colocalized with mitochondrial PDHE1α in merged image (white arrows). In Tg cells, PSD95 and PDHE1α expression was reduced in the neurites and mainly restricted to the soma, which was partially recovered by icariin treatment (white arrows); (**c**) Quantification of PSD95 and PDHE1α immunoreactivity showed a significant increase of PSD95 and PDHE1α expression in Tg + ICA neurons relative to Tg cells. Bar graphs (means ± SD) represented three independent experiments. * *p* < 0.05 *vs.* PSD95 level in NTg neurons; ^#^ *p* < 0.05 *vs.* PDHE1α level in NTg neurons; ^$^ *p* < 0.05 *vs.* PSD95 level in Tg neurons; ^@^ *p* < 0.05, *vs.* PDHE1α level in Tg neurons. Scale bars in (**b**) = 20 μm.

Mitochondrial morphology and distribution are maintained by mitochondrial fission/fusion proteins. The excessive fragmentation of mitochondria as observed in the present study may be associated with the abnormal mitochondrial dynamics in AD neurons. Here, we thus studied the effect of icariin on the expression of fission/fusion proteins in AD neurons by Western blotting. Our data showed that the expression level of mitochondrial fission protein dynamin-related protein 1 (Drp1) was significantly higher in Tg neurons relative to NTg cells, which was significantly decreased after treatment with icariin (Figure 4a). In contrast, the level of mitochondrial fusion protein mitofusin 2 (Mfn2) was lower in Tg neurons as compared to NTg controls, which was significantly increased by icariin treatment. These results suggest that icariin may preserve mitochondrial morphology and distribution through the modulation of mitochondrial fission and fusion in AD neurons.

Next, we sought to investigate the effects of icariin treatment on the expression of synaptic proteins in Tg hippocampal neurons and the association between synaptic protein expression and the mitochondrial distribution. Tg neurons were treated with icariin for 24 h and then double-labeling analysis for PSD95 and PDHE1α was performed. As shown in Figure 4b,c, NTg neurons exhibited intense staining of both PSD95 and PDHE1α in the cytosol and neurites, where they showed some colocalization. In Tg neurons, both the expression of PSD95 and PDHE1α was weaker in the neuronal processes and restricted to the somas, indicating that synaptic degeneration is associated with mitochondrial deficits. After icariin treatment, the PSD95 and PDHE1α expression was increased in the neuritic fibers of Tg cells. These data indicate that icariin may preserve key mitochondrial enzymes and synaptic functional proteins in AD-affected neurons.

### 2.5. Aβ Reduction Is Associated with Increased Pyruvate Dehydrogenase-E1α (PDHE1α) Expression in AD Neurons: Effect of Icariin

**Figure 5:**
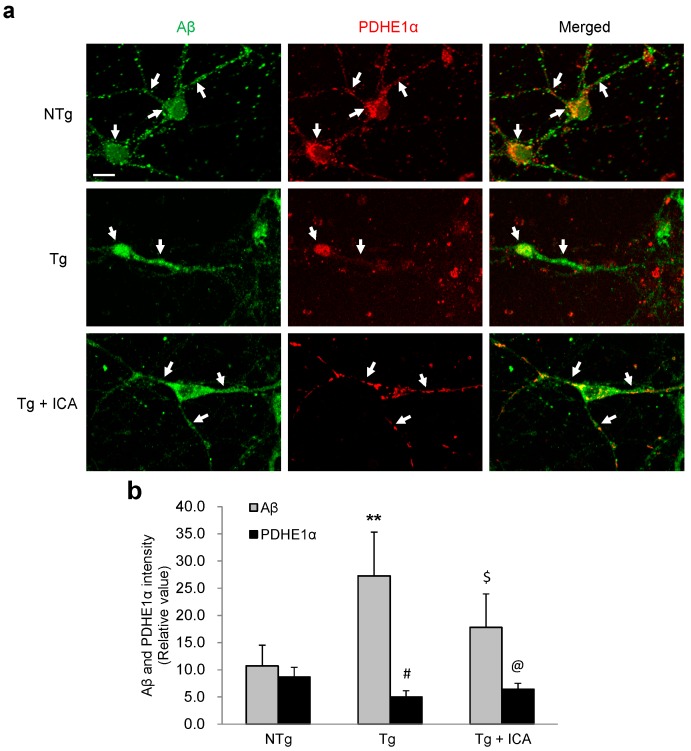
Double-labeling analysis of Aβ and PDHE1α in neurons treated with icariin. (**a**) NTg neurons exhibited a moderate labelling of Aβ with an abundant expression of PDHE1α in the cytoplasm and neurites (white arrows). In Tg cells the overexpression of Aβ in the soma and neurites was correlated with a weaker signal in PDHE1α staining (white arrows). Icariin-treated Tg cells showed increased PDHE1α expression in the neurites, accompanied with a downregulation of Aβ (white arrows); (**b**) Quantification of Aβ and PDHE1α immunoreactivity showed a higher expression of Aβ and a lower expression of PDHE1α in Tg neurons relative to NTg cells, which was reversed by treatment with icariin. Bar graphs (means ± SD) represented three independent experiments. ** *p* < 0.01 *vs.* Aβ level in NTg neurons; ^#^ *p* < 0.05 *vs.* PDHE1α level in NTg neurons; ^$^ *p* < 0.05 *vs.* Aβ level in Tg neurons; ^@^ *p* < 0.05, *vs.* PDHE1α level in Tg neurons . Scale bars in (**a**) = 20 μm.

To determine whether the protective effect of icariin on mitochondrial distribution in neurites was associated with the modulation of Aβ in Tg neurons, we performed double immunofluorescence staining for Aβ and the mitochondrial marker protein PDHE1α in primary neuronal cultures (Figure 5). NTg neurons exhibited moderate Aβ immunoreactivity in the cytoplasm and neurites, while PDHE1α expression was enriched in the cytosol and showed some colocalization with Aβ. In Tg cells, Aβ expression level was high in the cell bodies and neurites, whereas PDHE1α staining was much fainter in these areas. After icariin treatment, PDHE1α expression was increased in the cell bodies and neurites, which was accompanied by a downregulation of Aβ in the neurons derived from Tg mice. These results indicate that Aβ overexpression is associated with the abnormal distribution of mitochondrial proteins, and that icariin might be able to preserve mitochondrial protein expression by inhibiting the aberrant distribution of Aβ in Tg neuronal cells.

### 2.6. Effects of Icariin on Hyperphosphorylated Tau Expression in AD Neurons

**Figure 6:**
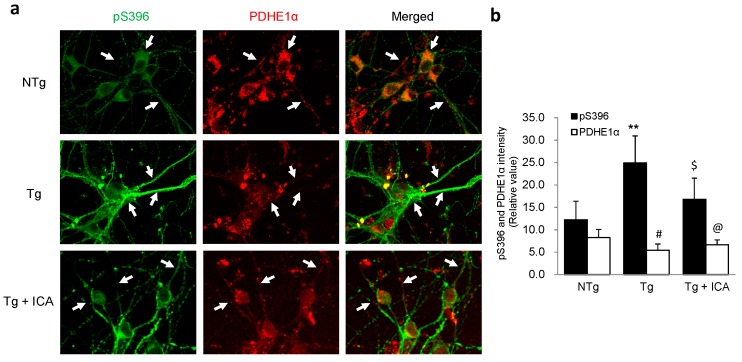
Double-labeling analysis of phosphorylated tau pS396 and PDHE1α in neurons treated with icariin. (**a**) NTg neurons showed a faint staining of pS396 in the neurites where PDHE1α expression was abundant (white arrows). In Tg cells, the strong pS396 staining was correlated with a weaker signal of PDHE1α immunoreactivity (white arrows). In icariin-treated Tg cells, the expression of PDHE1α was increased in the neurites, accompanied with a downregulation of pS396 (white arrows); (**b**) Quantification of pS396 and PDHE1α immunoreactivity showed a higher expression of pS396 and a lower expression of PDHE1α in Tg neurons relative to NTg cells, which was reversed by treatment with icariin. Bar graphs (means ± SD) represented three independent experiments. ** *p* < 0.01 *vs.* pS396 level in NTg neurons; ^#^ *p* < 0.05 *vs.* PDHE1α level in NTg neurons; ^$^ *p* < 0.05 *vs.* pS396 level in Tg neurons; ^@^ *p* < 0.05, *vs.* PDHE1α level in Tg neurons. Scale bars in (**a**) = 30 μm.

Hyperphosphorylation of tau protein is believed to cause abnormal mitochondrial distribution in AD diseased neurons. Thus, we further studied the effects of icariin treatment on phosphorylated tau expression by immunofluorescence analysis of Ptau-pS396 antibody with PDHE1α staining. As shown in Figure 6, pS396 immunoreactivity was higher in the neuritic fibers of Tg neurons relative to NTg cells, accompanied with a lower expression of PDHE1α in the corresponding areas. The reduction of pS396 staining in the neuronal processes of icariin-treated Tg cells was correlated with a stronger PDHE1α immunoreactivity in the neurites. These data reveal an inhibitory effect of icariin on tau protein hyperphosphorylation in AD affected neurons, which is consistent with previous publications [30].

## 3. Discussion

In this study, we investigated the protective effects of icariin on mitochondrial distribution and trafficking in hippocampal neurons from 3× Tg-AD mice. We observed decreased motility in axonal mitochondria, especially in anterogradely moving mitochondria in hippocampal neruons from 3× Tg-AD mice. The average length and size of the mitochondria was remarkably reduced in AD neurons, which is suggestive of increased fragmentation in axonal mitochondria. Our study demonstrated the first evidence that icariin could promote mitochondrial transport in the neuronal processes and partially rescue mitochondrial fragmentation in hippocampal neurons from 3× Tg-AD mice.

Normal transport of mitochondria in axonal processes is essential for providing energy to support synaptic activities such as neurotransmission and neuronal communication. Impairment in mitochondrial trafficking compromises synaptic function and causes neurodegeneration [23,24,25,26]. Thus, mitochondrial deficits are usually associated with synaptic dysfunctions in neurodegenerative diseases including AD. In this study, we found that mitochondrial movement is impaired in neurons from 3× Tg-AD mice, which is consistent with previous reports showing decreased mitochondrial motility in the Aβ-treated neurons [3,6,35]. Aβ has been found in mitochondrial membranes and is known to interact with mitochondrial proteins and enzymes, eventually leading to mitochondrial dysfunction [36]. Using *in vivo* live imaging analysis, severe mitochondrial impairments including the occurrence of mitochondrial fragmentation and decreases in the mitochondrial number and membrane potential were detected in the vicinity of Aβ plaque [5]. Our results also showed a dramatic reduction in the number of mitochondria in hippocampal neurons of 3× Tg-AD mice. In addition, the length and size of mitochondria were significantly decreased in AD neurons compared to in normal controls, indicating an increase in mitochondrial fragmentation and a shift in mitochondrial dynamics toward fission. Indeed, we observed a higher level of mitochondrial fission protein Drp1 in AD affected neurons, accompanied with a lower level of mitochondrial fusion protein Mfn2. The increase in mitochondrial fission and a decrease in fusion are associated with excessive mitochondrial fragmentation which leads to deficiencies in mitochondrial functions that may ultimately impair energy supply at nerve terminals. Our immunocytochemistry of primary hippocampal neurons revealed fragmented and punctuated immunoreactivity of key mitochondrial enzyme PDHE1α in the neuronal processes of AD neurons, further confirming the presence of mitochondrial fragmentation. It has been previously reported that mitochondrial dynamics are altered in neurons exposed to Aβ [37,38,39,40]. Mitochondrial accumulation of Aβ activates the expression of fission proteins Drp1 and Fis1, meanwhile reducing the expression of fusion proteins such as Mfn1, Mfn2 and Opa1 [37,38]. Our results showed that icariin treatment significantly reduced the level of Drp1 and increased the level of Mfn2 in AD neurons, which may be partially attributed to its inhibitory effect on the aberrant distribution of Aβ in AD neurons.

As a major pathological feature of neurodegenerative diseases such as AD, tau aggregation is also believed to have participated in the disruption of mitochondrial functions [22]. Hyperphosphorylation of tau protein has been shown to inhibit mitochondrial transport in the neurites of PC12 cells as well as the axonal processes of mouse brain cortical neurons, which may be attributed to the conformational changes in the microtubule spacing [21]. Additionally, tau may inhibit axonal transport of mitochondria through activation of axonal protein phosphatase 1 and glycogen synthase kinase 3, which is independent of microtubule binding [41]. Moreover, the trapping of the kinesin adaptor molecule JIP1 by phosphorylated tau may prevent JIP1 from loading mitochondria onto the kinesin machinery for anterograde transport along the axons [42]. The abnormal expression of tau may alter mitochondrial distribution, disrupt fission/fusion activity, increase oxidative stress, and cause a depletion of mitochondrial membrane potential [19,20,43]. In the present study, we found that the excessive expression of phosphorylated tau pS396 was correlated with a weaker expression of mitochondrial PDHE1α in AD neurons, which could be reversed by icariin treatment. Thus, our results support a role of icariin in the inhibition of tau protein hyperphosphorylation in AD affected neurons.

Icariin has been shown to improve the learning and memory abilities in Aβ25-35-induced AD rats or other AD animal models. Studies by Urano *et al.* showed that icariin improved spatial memory impairment in 5× FAD mice and recovered amyloid β (Aβ)-induced neuritic atrophy by extending axon and dendrite lengths in Aβ (1–42) treated rat cortical neurons [29]. Zeng *et al.* reported that icariin might exert its neuroprotective effects by inhibiting the Aβ-induced hyperphosphorylation of the tau protein, which likely occurs as a result of inhibiting glycogen synthase kinase-3β by activating the PI3K/Akt signalling pathway [30]. These results suggested an inhibitory effect of icariin in Aβ deposition and tau protein hyperphosphorylation. However, reports on the effects of icariin in sustaining mitochondrial functions are quite limited, particularly regarding mitochondrial trafficking and distribution in AD-affected neurons. Our previous studies showed that icariin administration improved cognitive functions and enhanced neurometabolite NAA level and ATP production in the hippocampus of 3× Tg-AD mice [32]. As a biomarker for neuronal integrity, NAA is synthesised in mitochondria and its level reflects mitochondrial energy state [44]. The preservation of key mitochondrial enzyme PDHE1α by icariin treatment might promote aerobic glycolysis in AD neurons and trigger the generation of acetyl-CoA, which is a substrate for NAA synthesis. Furthermore, the maintenance of mitochondrial transport in the axonal processes of AD neurons might enable the production of sufficient ATP at nerve terminals and fuel synaptic functions. As an effective component of traditional Chinese herbal medicine Epimedium, icariin functions as a phytoestrogen and displays estrogenic activity *in vivo* [45]. Estrogen is a crucial hormone within the metabolic system. Apart from its role in promoting female sexual development and reproductive capability, estrogen is also involved in multiple cellular events including the regulation of bone, immune, nervous and cardiovascular systems [10]. It is demonstrated that estrogen therapy can preserve glucose metabolism and has beneficial effects on cognitive function in postmenopausal women and thus reduces the risk of AD [46,47,48]. In the brain, estrogen receptors ERα and ERβ are widely distributed and have been found to be located in the nucleus and mitochondria, or embedded in membranes. The membrane-embedded estrogen receptors are involved in the activation of multiple intracellular signaling pathways such as Ras/Raf/MEKK/ERK, MAPK, PKC, Akt and PI3K/Src/ERK/CREB cascade and mediate neuronal survival and neuroprotection [10]. In mitochondria, estrogen receptors are found to directly bind mitochondrial DNA (mtDNA) and regulate mtDNA transcription. In the hippocampus, estrogen receptors have been demonstrated to locate the dendritic spines and take part in the regulation of synaptic protein expression. Thus, the protective effect of icariin on the preservation of mitochondrial and synaptic protein expressions as observed in this study is possibly related to its estrogen-like activities. Further study showed that icariin treatment resulted in a decrease in mitochondrial fission protein Drp1 and an increase in fusion protein Mfn2, implying that icariin may be able to maintain the normal shape and structure of mitochondria through the modulation of mitochondrial dynamics. As the first-step research work of icariin effect on mitochondrial transport, this study is limited to the level of the primary neuronal cultures, and further investigation of the *in vivo* model is necessary to address the mechanistic and functional connections between icariin and mitochondrial distribution at synapses.

Collectively, the data presented herein demonstrated that icariin could promote mitochondrial transport, protect mitochondria against fragmentation and preserve the expression of mitochondrial and synaptic functional proteins in primary hippocampal neurons from 3× Tg-AD mice. Icariin may exert its protective effects on mitochondria through the modulation of mitochondrial dynamics and the inhibition of aberrant Aβ and phosphorylated tau distribution in the diseased neurons. Our results suggest that icariin may be a potential therapeutic complement for AD and other mitochondrial malfunction-related neuronal degenerative diseases.

## 4. Experimental Section

### 4.1. Ethics Statement

This study was carried out in compliance with the Animal Care and Institutional Ethical Guidelines in China. All animal experiments were approved by the Ethic Committee of Shenzhen University (certificate number: SYXK 2014-0140).

### 4.2. Materials

Icariin was from National Institute for the Control of Pharmaceutical and Biological Products in China (Beijing) and dissolved in DMSO. Neurobasal medium and B27 supplement were from Invitrogen (Carlsbad, CA, USA). Penicillin-streptomycin, poly-d-lysine, DMSO and Triton X-100 were purchased from Sigma-Aldrich (St. Louis, MO, USA). Protease inhibitor cocktail was purchased from Roche Life Science (Shanghai, China). Aβ_1–42_ ELISA kit was purchased from Beijing Airan Technology Co., Ltd. (Beijing, China). pDsRed2-mito was from Clontech (Palo Alto, CA, USA). Lipofectamine 2000 was from Invitrogen (Carlsbad, CA, USA). The 35-mm Petri dishes were from MatTek Corporation. Antibodies against Aβ_1–42_, MAP2, PSD95, Mfn2, β-actin, VDAC and β-tublin were purchased from Abcam (Cambridge, MA, USA). The antibodies of PDHE1α and Drp1 were purchased from Santa Cruz Biotechnology (Santa Cruz, CA, USA). tau 46 was purchased from Cell Signaling Technology (Rockford, IL, USA). Tau-pS396 was purchased from Epitomics (Burlingame, CA, USA).

### 4.3. Neuronal Culture and Treatment

Cultures of primary hippocampal neurons were prepared from postnatal (P0–P1) pups of the triple-transgenic (3× Tg-AD) mice as described previously [32]. The nontransgenic (NTg) mice with the same genome background were used as normal control. 3× Tg-AD mice expressing mutant human transgenes *APP* (SWE), *PS1* (M146V), and *Tau* (P301L) were obtained from Jackson Laboratory (BarHarbor, ME, USA). Neuronal cultures were maintained in neurobasal medium with 2% B27 supplement. To examine the protective effect of icariin against mitochondrial dysfunction, primary neuronal cells were treated with 20 μM icariin for 24 h at day 10 *in vitro* (10 DIV).

### 4.4. Immunocytochemistry Assay

For immunocytochemistry, NTg and Tg cells were fixed with 4% paraformaldehyde for 20 min, permeabilized with 0.2% Triton X-100 for 15 min, blocked with 10% goat serum in PBS for 1 h, and then incubated with primary antibodies including Aβ_1–42_ (1:200), PDHE1α (1:100), PSD95 (1:1000), tau 46 (1:500), Tau-pS396 (1:200), and MAP2 (1:8000) overnight at 4 °C, followed by secondary antibodies labeled with Dylight-488 or Dylight-594 for 1 h. Cells were visualized using an inverted laser-scanning confocal microscope (OLYMPUS FV1000, Tokyo, Japan). Image analysis was performed with ImageJ software (NIH, Bethesda, MD, USA).

### 4.5. Western Blot Assay

For Western blot assay, cells were lysed in 1× lysis buffer supplemented with protease inhibitor cocktail according to the manufacturer’s instructions. The protein concentration was measured by the BCA protein assay kit (Beyotime Institute of Biotechnology, Shanghai, China). Then, total cell lysates (20 μg per well) for each sample was separated by SDS-PAGE and transferred to a polyvinylidene fluoride (PVDF) membrane (Millipore, Billerica, MA, USA). Membranes were blocked with 5% fat free milk before incubation with primary antibodies (1:1000) at 4 °C overnight, followed by incubation with the corresponding HRP-labeled anti-rabbit or anti-mouse secondary antibodies at room temperature for 1 h. The bound antibodies were detected with ECL reagent and visualized by KODAK Image Station 4000 MM (Carestream Health Inc., New Haven, CT, USA). The density of protein bands was measured using Quantity One software (Bio-Rad, Hercules, CA, USA).

### 4.6. Enzyme Linked Immunosorbent Assay (ELISA)

Aβ levels were measured in the cells and medium collected from NTg and Tg neuronal cultures using an ELISA kit of Aβ_1–42_ (Beijing Airan Technology Co., Ltd., Beijing, China).

### 4.7. Mitochondrial Trafficking Recording and Data Analysis

Axonal mitochondria were visualized by the transfection of pDsRed2-mito in hippocampal neurons at 8 DIV using lipofectamine 2000 according to the manufacturer’s protocol. Tg neurons were treated with 20 μM icariin at 10 DIV and then imaged 24 h later. Axons were identified by morphological characteristics. Processes extending from the soma that are two to three times longer than other fibers were considered to be axonal fibers. Time-lapse images were taken every 5 s for a total of 2 min under 60× magnification, using an inverted OLYMPUS FV1000 confocal microscope with a stage-based chamber (5% CO_2_, 37 °C). Kymographic images showing the overall trafficking of mitochondria were generated by using ImageJ software with a Mulitple Kymograph plug-in. Mitochondrial movement data were analyzed from the kymographs. Mitochondria was considered to be nonmobile if the displacement was no more than 2 μm for the entire recording period. At least 10 samples were collected for each condition and scored.

### 4.8. Axonal Mitochondrial Content Measurements

Following time-lapse imaging of the mitochondria in primary hippocampal cultures, neurons were fixed in 4% paraformaldehyde for 20 min and then imaged under an inverted OLYMPUS FV1000 confocal microscope with a 60× objective. DsRed-mito labeled mitochondria were analyzed by means of the ImageJ program to evaluate the axonal mitochondrial index, mitochondrial length, and the density of mitochondria. The experiments were repeated three times with at least four cells each culture for each condition.

### 4.9. Statistical Analysis

The statistical difference was analyzed using Student’s *t*-test and analysis of variance (ANOVA). All data were presented as mean ± standard deviation (SD).

## Supplementary Materials

Supplementary materials can be found at http://www.mdpi.com/1422-0067/17/2/163/s1.
